# Random Copolyester-Based Delivery Systems for Tear Protein Therapeutics in Ocular Surface Disorders

**DOI:** 10.1021/acsomega.5c09194

**Published:** 2026-05-18

**Authors:** Gloria Astolfi, Giulia Guidotti, Michelina Soccio, Franco Dominici, Debora Puglia, Nadia Lotti, Erika Ponzini, Silvia Tavazzi, Piera Versura, Luigi Fontana

**Affiliations:** † Ophthalmology Unit, DIMEC, 9296 Alma Mater Studiorum University of Bologna, Bologna 40138, Italy; ‡ Department of Civil, Chemical, Environmental and Materials Engineering, Alma Mater Studiorum Università di Bologna, Bologna 40131, Italy; § Department of Civil and Environmental Engineering, 9309 University of Perugia, 05100 Terni, Italy; ∥ Department of Materials Science, University of Milano-Bicocca, 20126 Milan, Italy; ⊥ IRCCS Azienda Ospedaliero-Universitaria di Bologna, Bologna 40138, Italy

## Abstract

This study presents a polymeric drug delivery platform designed to address the limitations of conventional therapies for ocular surface and corneal disorders, such as dry eye disease (DED). Conventional treatments typically require frequent administration of eye drops, leading to poor bioavailability and patient adherence. We investigated the use of two random poly­(butylene succinate/diglycolate) copolymers, namely, P­(BS_*x*_BDG_*y*_) at varying molar ratios (*x*, *y*) as vehicles for conjunctival drug delivery. Copolymers were synthesized and extensively characterized by their molecular structure, thermal and mechanical properties, and surface wettability. Prototype cylindrical devices (2 × 3 mm) were fabricated by microinjection molding. Lysozyme (Lys) and lactoferrin (Lf), two endogenous tear proteins with therapeutic relevance, were loaded into the polymer matrices, and their release profiles were evaluated. *In vitro* cytotoxicity assays confirmed the biocompatibility of the materials, supporting cell viability over 24 h. Protein release studies demonstrated a sustained release from the copolymeric matrices, with both proteins retaining structural integrity throughout the release period. These findings indicate that P­(BS_50_BDG_50_) and P­(BS_20_BDG_80_) based devices exhibit key features suitable for ocular surface drug delivery: structural adaptability, controlled release kinetics, and compatibility with biologically active macromolecules. These platforms may represent a promising tool for targeted and prolonged drug administration in ocular diseases requiring frequent and localized treatment.

## Graphical abstract

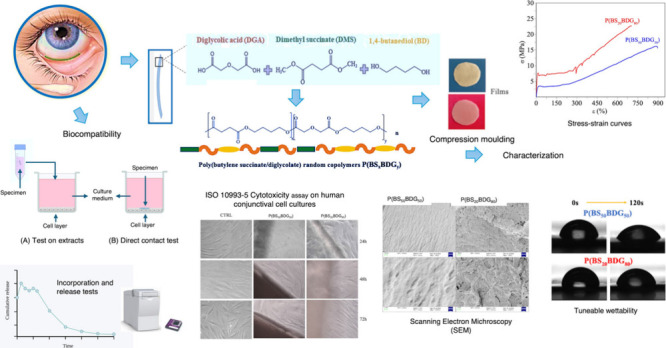

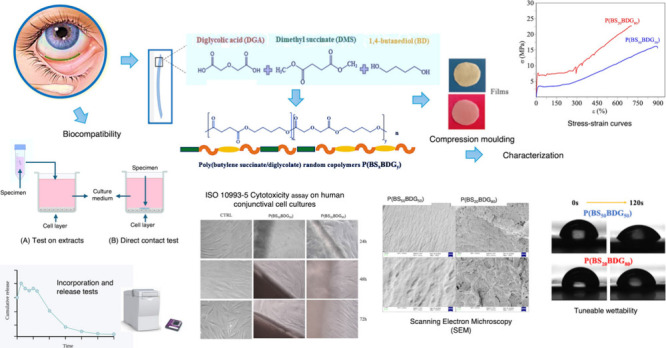

## 1 Introduction

Advances in biomedical engineering have triggered the emergence of drug delivery systems that seek to address the shortcomings of conventional therapies, particularly with respect to chronic disorders that require frequent dosing.^1^ Standard dosing regimens typically lead to periods of unintended peak concentrations above toxic levels and subsequent periods of below-effective concentrations.^2^ This variability is a consequence of short time to peak of drug and clearance rates, which results in under- or overtreatment.^3^ Localized and controlled drug delivery systems provide significant opportunities to improve therapeutic efficacy and limit systemic exposure and side effects.^4^ The ocular surface is an especially challenging target for drug delivery because of anatomical and physiological barriers that impact the therapeutic performance of drugs topically administered. These include eye blink and subsequent tear turnover of drug and nasolacrimal drainage, which limit both retention and permeation of drugs applied to the ocular surface.^5^ Dry eye disease (DED) is among the most common ocular surface diseases and especially impactful to eye health.^6,7^ The *Tear Film & Ocular Surface Society* (TFOS) defines DED as a multifactorial disease of the ocular surface characterized by loss of homeostasis of tear film stability, hyperosmolarity, inflammation, and damage to the ocular surface and associated symptoms of discomfort and visual disturbance.^8^ The impact of DED is particularly prevalent among older adults and women and in the presence of other concurrent conditions such as diabetes mellitus and ceramic exposure.^9^ Despite its significant impact on quality of life, this disease remains significantly underdiagnosed and undertreated, leading to further economic impact and loss of productivity.^10^ Ophthalmic drug products are predominantly topical formulations, such as eye drops, accounting for nearly 90% of marketed ophthalmic products.^11^

Despite having such a widespread applicability, the ocular bioavailability of medicines administered topically remains fairly low and, typically, less than 5% of a topical medicine reaches anterior ocular tissues because of the rapid elimination from the precorneal area.^5,12^ Therefore, alternative drug delivery systems (DDSs) have been investigated to improve the ocular bioavailability of medications and to enhance ocular medicine efficacy.^12–14^ In this context, polymeric materials have emerged as a potential solution in the field of ocular drug delivery.^15–18^ Indeed, thanks to the high availability of monomers, they can be properly designed according to this specific application, and they can also be processed into systems capable of overcoming the eye barrier and improving the release profile.^19,20^ The success of these materials is also confirmed by market data of polymers for drug delivery, which was of about USD 1,749 Billion in 2023 and is predicted to reach USD 3,165 Billion from 2024 to 2031.^21^

To date, the most used synthetic polymeric materials for ocular applications are poly­(ethylene glycol) (PEG), poly­(vinyl alcohol) (PVA), poly­(lactic acid) (PLA), poly­(glycolic acid) (PGA), poly­(lactic-*co*-glycolic acid) (PLGA), poly­(caprolactone) (PCL), polymethacrylates such as poly­(2-hydroxyethyl methacrylate) (HEMA), and polyolefins like poly­(acrylic acid) (PAA).^20,22^ Among this wide family of polymeric materials, polyesters and, in particular, biodegradable ones, turned out to be particularly promising, as they allow site-specific, tunable degradation kinetics aligned with the intended drug release profile.^23–25^ Last but not least, biodegradable polyesters are usually biocompatible, a necessary requirement for any application in contact with human tissues. Accordingly, ocular DDSs based on biodegradable polymers have been developed to increase drug residence time on the ocular surface and obtain a controlled delivery to facilitate drug penetration to ocular tissues.^20^ In particular, these materials have been widely employed in the development of nanoparticles, hydrogels, inserts, micelles, and in situ gelling formulations for anterior segment diseases due to their biocompatibility, established processing methods, and regulatory familiarity.^16,26–28^ Some commercially available examples include Ozurdex and Durysta, which are devices made of a PLGA matrix, used for the ocular release of dexamethasone and bimatoprost, respectively.^29,30^ Although PLA-, PLGA-, and PCL-based systems are widely employed in ocular drug delivery, several limitations remain. Beyond burst release and limited long-term release control, polyester-based materials such as PLGA may generate acidic degradation byproducts, potentially affecting protein stability and local tolerability.^12^ Additionally, variability in degradation kinetics, processing constraints incompatible with sensitive biomolecules, and suboptimal mechanical adaptability or surface wettability may compromise retention, comfort, and interaction with the tear film.^14,26,31^ Moreover, PLA, PGA, and their copolymers are particularly stiff materials, with elastic moduli exceeding 1 GPa.^32,33^ In contrast, human ocular tissues are significantly softer, with elastic moduli in the range of 0.1 to 3 MPa.^34^ This difference may induce the sensation of a foreign body, with high discomfort for the patient and a risk of an inflammatory response. On the other hand, PEG-based materials, for example, show a too rapid erosion in physiological environment,^26^ resulting in limited stability over time.

For all these reasons, a single biomaterial is often unluckily to satisfy alone all the requirements for a specific use, including thermomechanical properties, easy processing, and the proper release profile, thus limiting its real application.

Therefore, the design of materials with *ad hoc* properties is of primary importance to provide the maximum comfort to the patient, ensuring the long-term success of the device. To this aim, copolymerization is a tool widely used to chemically modify a polymer in order to obtain an optimal set of properties for the intended application.^35,36^

According to the aforementioned scenario, in the present study we detail the design, synthesis, and characterization of an ocular DDS made with random aliphatic copolyesters of PBS and PBDG.

These two biocompatible homopolymers have been already investigated in the literature for biomedical purposes: PBS, which is already commercially available^37,38^ for packaging and agricultural films, biodegradable bags, single-use food containers, and cutlery, can find further applications in biomedicine, in the fields of tissue engineering, controlled drug delivery, and to realize bioresorbable devices.^39–41^ Conversely, diglycolic acid is often employed as a comonomeric unit to improve surface wettability, mechanical flexibility, and degradation rate^42,43^ of the final copolymer. However, to the best of our knowledge, the use of systems based on these materials in ocular drug delivery has not yet been reported. Moreover, succinic acid, one of the starting monomers, can be found in the inactive ingredient database of the United States Food and Drug Administration (USFDA), as it is already employed in the form of gel, tablet, and capsule for oral, topical, and intravenous delivery.^44^

The materials were synthesized by two-step melt polycondensation, which is a green, solvent-free technique,^45^ already employed at industrial level and particularly safe in terms of applications in contact with human body, and first processed into flexible compression-molded films. Characterization was performed through molecular, thermal, mechanical surface, and cytotoxicity analyses on the films. Given their thermal stability and easy processability, a cylindrical prototype device was also fabricated by extrusion and evaluated for protein encapsulation and *in vitro* release. The proposed copolymeric drug delivery system therefore represents a rationally engineered approach that integrates tunable material properties with controlled release, offering a versatile strategy for ocular-surface drug delivery.

## 2 Materials and Methods

### 2.1 Materials

Dimethylsuccinate (DMS), diglycolic acid (DGA), 1,4-butanediol (BD), titanium tetrabutoxide (Ti­(OBu)_4_), thiazolyl blue terazolium bromide (MTT), lysozyme (Lys), and lactoferrin (Lf) were obtained from Sigma-Aldrich. Wong Kilbourne derivative of the Chang (WKD) conjunctiva-derived epithelial cell line (clone 1-5c-4, American Type Culture Collection (ATCC)-certified cell line (CCL 20.2), Dulbecco’s Modified Eagle’s Medium (DMEM), fetal bovine serum (FBS), trypsin 0.25% 1 mM EDTA from Gibco, and antibiotic/antimycotic solution (penicillin 10.000 U/mL, 10.000 μg/mL) were purchased from Lonza Group (Ltd., Basel, Switzerland).

### 2.2 Synthesis and Characterization of Copolymers

Poly­(butylene succinate/diglycolate) random copolymers, P­(BS_*x*_BDG_*y*_), where *x* and *y* represent the relative molar amounts of the two counits) were synthesized in bulk by two-stage melt polycondensation (Figure 1), according to the procedure previously reported.^46^

**Figure 1:**
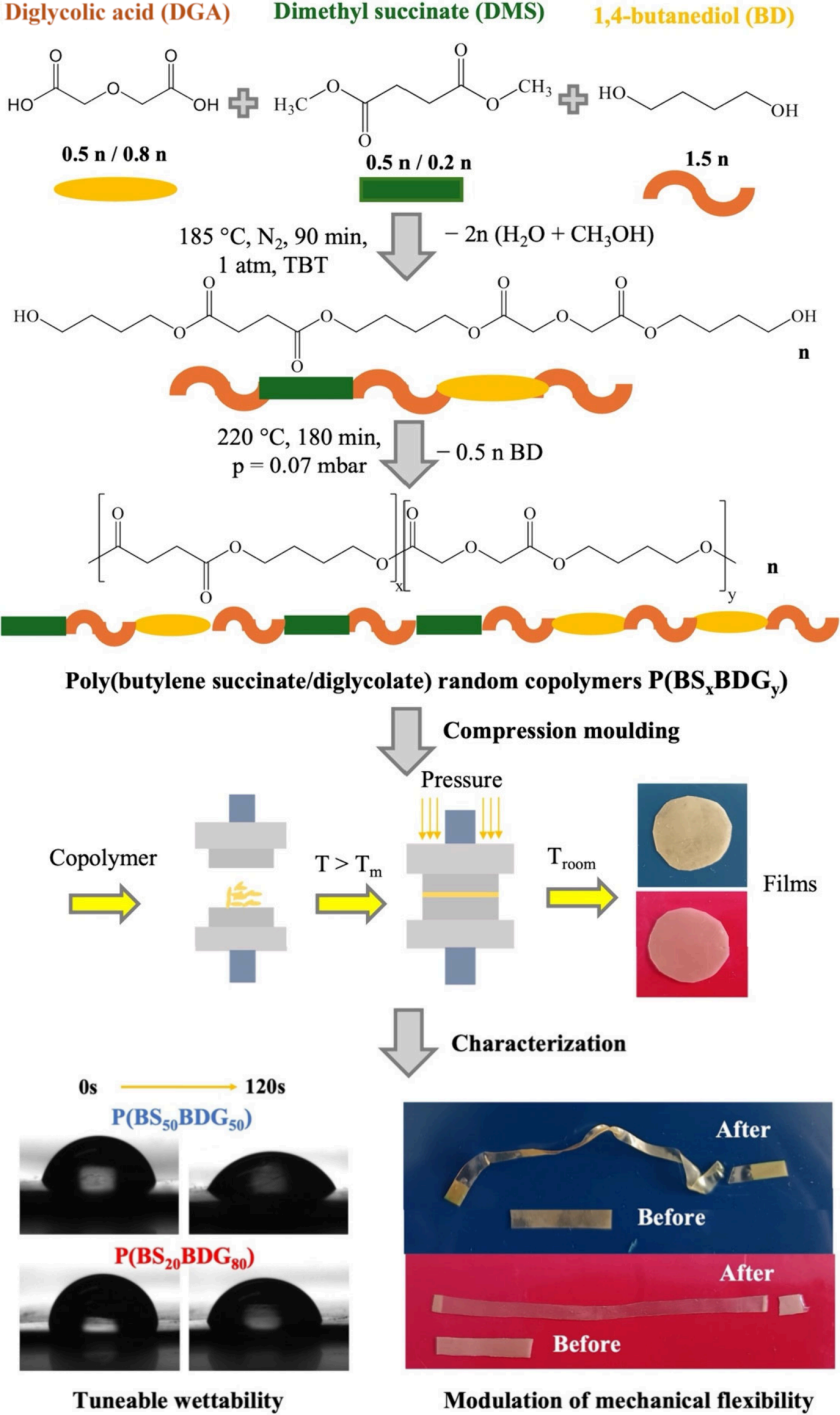
Schematic representation of the synthetic path, the compression molding processing, and the main functional properties of the copolymers obtained.

The obtained polymers were purified through dissolution in chloroform and consequent precipitation into a large excess of methanol and then processed into films (150 μm thick) by compression molding (Carver, C12).

Proton nuclear magnetic resonance (^1^H NMR, Varian Inova 400-MHz) was performed to confirm both the chemical structure and the chemical composition of the copolymers. To this aim, polymeric solutions (10 mg/mL) were obtained dissolving each material in deuterated chloroform with tetramethylsilane (TMS, 0.03 vol %) as internal standard. Gel permeation chromatography (GPC, HPLC 1100 equipped with a PLgel 5 mm MiniMIX-C column and a RI detector) was carried out to determine the molecular weight (*M*_n_) and the polydispersity index (Đ).

Thermogravimetric analysis (TGA, PerkinElmer TGA4000) was performed under pure N_2_ flow. Samples (5 mg) were heated at 10 °C/min from 40 to 800 °C, and the temperatures of initial decomposition (*T*_id_) and of maximum degradation rate (*T*_max_) were calculated from the obtained thermograms. Differential Scanning Calorimetry (DSC, PerkinElmer DSC6) was performed under pure N_2_ flow. Samples (8 mg) were subjected to the following thermal treatment: heating (20 °C/min) from −70 to 140 °C (I scan); holding for 3 min; rapid cooling (100 °C/min) to −70 °C; holding for 15 min; and heating (20 °C/min) to 140 °C (II scan).

Static contact angle (WCA) measurements were carried out on polymeric films, previously washed in an aqueous solution of ethanol (70 vol %), using a Krüss DSA30S instrument, by recording the side profiles of deionized water drops (4 μL, 100 μL/min). Mechanical tensile tests were carried out using an Instron 5966 dynamometer equipped with a 10 kN load cell. Film stripes (5 × 0.5 cm) with a gauge length of 2 cm, were stretched at a rate of 10 mm/min, and the values of tensile strength at break (σ_B_), elongation at break (ε_B_) and Young’s modulus (E) were calculated from the so-obtained stress–strain curves.

### 2.3 Extrusion of a Cylindrical Structure

The purified copolymers were used for extrusion tests in order to obtain cylindrical shapes with a diameter of 0.25 mm. The processing of the materials was carried out with a corotating twin-screw microextruder (DSM mod. Xplore 15 Microcompounder). The specimens were obtained thanks to a micro injection press (DSM mod. Micro 12 cm^3^ Injection Molding Machine). About 15–20 g of each material were loaded into an injector, and then extruded, resulting in a thread with a cylindrical shape. The adopted processing parameters are the following: screw speed 60 rpm, force 2500 N, mixing time 180 s, Heating of Front and rear (top 54 °C, middle 58 °C, bottom 60 °C, extrusion melt 59 °C).

### 2.4 Scanning Electron Microscopy (SEM)

The morphology of the fractured surfaces for each extruded cylinder was observed with a field-emission electronic microscope Supra 25 (Zeiss AG, Oberchochen, Germany).

### 2.5 Protein Elution Experiments

Protein elution analyses from each polymer were performed by using two model proteins: lysozyme (Lys) and lactoferrin (Lf). The molecules were encapsulated in three different ways: by melting the copolymers with the molecules, by placing the copolymers within protein solutions, and by encapsulating the proteins within a coating outside the cylinder. Quantification of protein concentrations was assessed with a kit associated with the Bioanalyzer 2100 instrument (Agilent).

#### 2.5.1 Loading of Model Proteins

First, specific amounts of polymer and protein were weighed to obtain concentrations of 10 mg/g. The encapsulation of the protein within the polymer matrix was carried out by means of a minimixer setting the chamber at 60 °C for 5 min, to prevent proteins denaturation. Films were cut into discs (approximately 1 cm^2^ area), immersed in 1× phosphate buffered saline (PBS) pH 7.4, and aliquots were taken at different times in order to analyze the protein release kinetics. Subsequently, model proteins were loaded by immersion: discs of both copolymers, obtained from their respective films, were loaded by immersion with 3 mg/mL Lys and Lf respectively, individually suspended in PBS 1x for 3h. Last, model proteins were dispersed into coated cylinders. Each polymer (PVA 20:1, PVP K-30 2:1 and agar 1.5%) was employed as a coating for cylinders and loaded with Lys and Lf (final concentrations of 3 mg/mL for Lys and 2.5 mg/mL for Lf, respectively) and then fully dried. To evaluate the elution curve, aliquots were taken at different time points and then quantified.

### 2.6 Circular Dichroism

Cylindrical samples were incubated for 4 h in a sterile 0.9% NaCl solution containing Lys and Lf (3 mg/mL each), approximating their tear fluid concentrations. After drying, samples were transferred to fresh sterile 0.9% NaCl for release testing. Following 3 h of incubation, the release medium was placed in a 1 cm quartz cuvette for circular dichroism (CD) analysis using a Jasco J-815 spectropolarimeter (JASCO Corp., USA). CD spectra of the protein solutions were also recorded prior to the loading. Measurements were performed from 206 to 260 nm with a 0.5 nm bandwidth, a 0.25 nm data pitch, and a 0.5 s response time.

### 2.7 Biocompatibility Evaluation

Cytotoxicity tests were performed according to the experimental procedures described in ISO 10993-5.^47^

#### 2.7.1 Cell Culture

Two cytotypes were obtained from two conjunctival tissues: primary human conjunctival fibroblasts (HConF) were derived from normal human conjunctiva, while the Wong Kilbourne were derived from the Chang (WKD) conjunctiva-derived epithelial cell line (ATCC), (HConEpiC). Cells were maintained at 37 °C in a humidified 5% CO_2_ in Dulbecco’s Modified Eagle’s Medium (DMEM) with glutaMAX culture medium (from Gibco) supplemented with 10% fetal bovine serum (FBS, Gibco), 1% antibiotic, antimycotic solution (penicillin 10.000 U/mL, 10.000 μg/mL, Lonza Group Ltd., Basel, Switzerland). Cells from passages 1 through 5 were used. HConEpiC from passages 15 through 30 (after ATCC initial passage 65) were used in all experiments. The normal culture development, morphology, and level of confluence were assessed daily by observation under an inverted light microscope. Reaching 80% confluence, cells were detached with trypsin 0.25% containing 1 mM EDTA (Gibco) and counted prior to seed into culture microplates.

#### 2.7.2 Cytotoxicity Tests

Circular polymeric films (1 cm diameter) were first prewashed with 90% v/v ethanol for 30 min and 70% v/v ethanol for 30 min and then washed 3 times with fresh culture medium prior to further assay. Cells were seeded in 24-well culture plates at a density of 2 × 10^4^ cells per well in 0.5 mL of complete medium in order to assess the direct contact test and into 96-well culture dishes at a density of 1.5 × 10^4^ cells per well to assess extract test, respectively, and kept at 37 °C for 24 h.

Direct contact tests were performed by replacing the culture medium after a 24 h incubation with medium prepared by adding 5% FBS. Circular polymeric films were immersed within each well and incubated under the same conditions for up to 72 h. For the control test, a well was seeded under the same conditions without adding any material. Tests were also performed on extracts, employing an extraction ratio of 10 mg/mL^–1^. The extract test was performed in accordance with ISO 10993-5 and 10993-12 ^47,48^ guidelines for sample preparation and extraction of medical device materials. Copolymer films were cut into small rectangular pieces (approximately 10 mm × 50 mm) to enhance complete immersion in the extraction medium. An extraction ratio corresponding to approximately 6 cm^2^ of material surface per 1 mL of complete culture medium was employed, resulting in an effective extraction concentration of 10 mg/mL. Material samples were incubated in complete culture medium (DMEM supplemented with 5% FBS) for 24 h at 37 °C to obtain the extraction media. The resulting extracts were used as 100% concentration and subsequently serially diluted with complete medium to obtain final extract concentrations of 50% and 25% (v/v), in accordance with the logarithmic dilution range recommended by ISO 10993-5. After 24 h of cell seeding, the culture medium was removed and replaced with the prepared extraction media. Cells were then incubated for an additional 24, 48, and 72 h prior to viability assessment.

#### 2.7.3 MTT Assay

As outlined in ISO 10993-5, MTT is resuspended fresh in MEM without supplements at a final concentration of 1 mg/mL. The solution was added to each sample, followed by an incubation of 24 h at 37 °C, under a CO_2_ (5%) atmosphere. The day after incubation the supernatant was removed and then 100 μL of isopropanol was added. The plate was gently swayed for a few minutes and absorbance was measured at 570 nm using a Sky High Multiskan microplate reader (Thermo-Fisher Scientific) at a reference wavelength at 630 nm. The experiments were repeated at least three times with biological replicates and data are expressed as means value ± standard deviations.

## 3 Results and Discussion

### 3.1 Synthesis and Characterization of the Polymers

In the present study, two random copolymers with different compositions, P­(BS_50_BDG_50_) and P­(BS_20_BDG_80_), have been successfully prepared. These two compositions were properly chosen according to previous studies carried out by some of the authors.^46,49^ As a matter of fact, from the thermal point of view, they ensure a certain amount of crystalline domains, which is fundamental for any kind of processing, as well as a melting temperature high enough not to melt inside the human body. In parallel, the presence of ether oxygen atoms gives the final material high mechanical flexibility and surface wettability^46,49,50^ (Figure 1), necessary requirements for the intended application. The data concerning the molecular characterization of copolymers are reported in Table 1.

**Table 1:** Molecular (Gel Permeation Chromatography, GPC, and Proton Nuclear Magnetic Resonance, ^1^H-NMR), Thermal (Thermogravimetric Analysis, TGA, and Differential Scanning Calorimetry, DSC), Surface Wettability (Water Contact Angle, WCA), and Mechanical (Tensile Testing) Characterization Data of P­(BS_50_BDG_50_) and P­(BS_20_BDG_80_) Copolymers Investigated in This Work, Compared to Those of the Reference Homopolymers

			P(BS_50_BDG_50_)	P(BS_20_BDG_80_)	PBS^a^	PBDG^a^
Molecular characterization	GPC	*M*_n_ (g/mol)	49800	46800	51200	28100
		Đ (−)	2.1	2.2	2.3	2.0
	^1^H NMR	BDG feed (mol %)	50	80	-	100
		BDG actual (mol %)	52	82	-	100
Thermal characterization	TGA	*T*_id_ (°C)	347	348	-	-
		*T*_max_ (°C)	377	381	395	380
	I scan DSC	*T*_g_ (°C)	–30	–27	–32	–23
		Δ*c*_p_ (J/(g °C))	0.515	0.303	0.101	0.296
		*T*_m_ (°C)	33	39	115	66
			39	49		
		Δ*H*_m_ (J/g)	20	42	60	55
	II scan DSC	*T*_g_ (°C)	–32	–27	–39	–27
		Δ*c*_p_ (J/(g °C))	0.549	0.595	0.105	0.672
		*T*_m_ (°C)	-	-	116	-
		Δ*H*_m_ (J/g)	-	-	64	-
Surface wettability	WCA	0 s (deg)	80 ± 1	91 ± 2		
		120 s (deg)	63 ± 1	78 ± 2		
Mechanical characterization	Tensile testing	*E* (MPa)	53 ± 4	143 ± 16	337 ± 26	148 ± 21
		σ_B_ (MPa)	15 ± 1	21 ± 3	31 ± 2	23 ± 2
		ε_B_ (%)	871 ± 61	655 ± 63	24 ± 4	427 ± 33

a Data from refs 49 and 51 .

In more detail, GPC measurements have been performed to determine the values of molecular weight (*M*_n_), which must be sufficiently high to ensure the proper workability of the materials. As can be noted, both materials show high and comparable values of *M*_n_, in line with that obtained for PBS and even higher than the one of PBDG (Table 1), with polydispersity indexes in all cases around 2, typical of polycondensation-derived polymers, indicating the optimization of synthetic conditions.

According to previous studies by some of the authors,^49^ ^1^H NMR spectra were found to be consistent with the expected structure, further confirming the proper control over polymerization. The ^1^H NMR spectra of the two copolymers are shown in Figure S1: apart from the chloroform signal at δ 7.26 ppm and the TMS signal at δ 0 ppm, only peaks relating to the protons of the different copolymers can be identified. The actual composition, calculated from the relative areas of the ^1^H NMR resonance peaks of the aliphatic protons of the succinic subunit located at 2.6 ppm and of the protons of the diglycolic subunit at 4.3 ppm (Figure S1), resulted in both cases like the feed one.

#### 3.1.1 Thermal Characterization

The *T*_id_ and *T*_max_ values obtained by thermogravimetric analysis are collected in Table 1, while the thermograms of the two copolymers are shown in Figure S2. In all cases, thermal stability was high and comparable, with *T*_id_ between 347 and 348 °C and *T*_max_ in the range of 377 and 381 °C, respectively, thus ensuring a wide processing window. These values are also similar to those of the reference homopolymers,^49^ indicating that copolymerization did not adversely affect this parameter. The two thermograms are characterized by a single-step degradation profile and a residual char of approximately 4% of the initial weight.

The copolymeric films were also subjected to calorimetric analysis (DSC), to evaluate their main thermal transition. As it can be seen (Table 1, Figure S3), both materials exhibit the typical profile of rubbery and semicrystalline materials, with *T*_g_ < *T*_room_ and a melting peak at higher temperature, respectively. The effect of copolymerization can be observed in a decrease in *T*_m_ and Δ*H*_m_ compared to the reference homopolymers (Table 1),^49^ due to the formation of a less perfect crystalline phase, present also in smaller quantities, respectively. The presence of less perfect crystals is also confirmed by the shape of the melting peaks, which are enlarged and consist of two overlapping endothermic phenomena, which can be attributed to melting-crystallization-melting of crystals.^52,53^ This effect depends also on the chemical composition: in fact, P­(BS_50_BDG_50_) is lower melting than P­(BS_20_BDG_80_), with a melting enthalpy value practically halved, indicating a greater suppression of crystallization.

In the II scan, which was carried out after rapid cooling from the melt, both materials turned out to be amorphous, since only the *T*_g_ jumps can be observed (Figure S3). Taking into account the *T*_g_ values of the two homopolymers (Table 1), the values measured for P­(BS_50_BDG_50_) and P­(BS_20_BDG_80_) are in line with their chemical composition and equal to −32 °C and −27 °C respectively. This predictable evidence indicates that the former material, which contains a higher amount of BS counits, is also characterized by a slightly higher chain mobility.

#### 3.1.2 Mechanical Characterization

Mechanical characterization data (elastic modulus, E, stress at break, σ_B_, and elongation at break, ε_Β_) of the test specimens are listed in Table 1, while the relative stress–strain curves are shown in Figure S4.

First of all, the effect of copolymerization can be noticed by comparing the mechanical data of the materials under study to those of the reference homopolymers. In both cases, a decrease in the elastic moduli can be observed, together with a remarkable improvement in the elongations at break (Table 1). As to the copolymers, P­(BS_20_BDG_80_) is the most rigid material, with an elastic modulus (E) of 143 MPa, whereas P­(BS_50_BDG_50_) shows an elastic modulus about 3 times lower (53 MPa). Since the molecular weights are comparable for both materials, as well as their *T*_g_ values, the differences in mechanical behavior must be attributed to the higher crystallinity of P­(BS_20_BDG_80_), compared to P­(BS_50_BDG_50_) (Table 1). For the same reason, an opposite trend can be observed for the values elongation at break (ε_Β_), even though in both cases it is particularly high, exceeding 650%. Of note, the mechanical characteristics of both the copolymers are suitable for the realization of devices in contact with eye. Indeed, the obtained values of elastic modulus are characteristic of relatively soft polymeric materials and remarkably lower than those of other aliphatic polyesters used in ophthalmology, such as PLA, PGA and PLGA,^54–56^ and are generally considered suitable for contact with soft tissues in non-load-bearing applications, while the high elongation and stress at break ensure good handling, which is necessary for proper device placement without compromising its integrity.^57^ Lastly, at elongation values below 20%, the yielding phenomenon is present (Figure S4).

### 3.2 Contact Angle Measurements (WCA)

The compression molded films were subjected to contact angle measurements (WCA). The evaluation of this parameter is of great importance, considering that the final application of these materials is in contact with a particularly humid environment, such as the eye. According to the values listed in Table 1 and to the pictures shown in Figure 1, immediately after deposition P­(BS_20_BDG_80_) turned out to be quite hydrophobic, while P­(BS_50_BDG_50_) was hydrophilic (WCA values of 91 and 80°, respectively). As known, the introduction of ether oxygen atoms in a macromolecular chain is responsible for an increase in surface wettability.^50,58^ However, this parameter cannot be the only factor to be considered, since the copolymer richer in DG counit is the most hydrophobic. Therefore, also the kind of crystalline phase developed, and, in turn, the type of counits rejected in the mobile amorphous phase, play a key role in determining the surface wettability.^13^ Indeed, P­(BS_50_BDG_50_) is a less-crystalline material, with a crystalline phase rich in PBS and amorphous domains richer in hydrophilic DG moieties. Conversely, in P­(BS_20_BDG_80_) copolymer, the crystalline phase developed is DG-rich, with an amorphous phase richer in the hydrophobic PBS.^46,49^

Last, after 2 min from the droplet deposition, the WCA values increase, of about 20° for P­(BS_50_BDG_50_) and of about 13° for P­(BS_20_BDG_80_), respectively. This result indicates that, after prolonged exposure to water, both copolymers can be considered hydrophilic. This behavior cannot be due to degradation of the polymeric films, as aliphatic polyesters containing ether-oxygen atoms are known to biodegrade upon longer time points and complete immersion in aqueous environment.^60^ Conversely, the results obtained indicate that, after prolonged exposure to water, the polymeric chains of both copolymers are able to rearrange at the surface, making the materials hydrophilic.

From a translational perspective, the higher Young’s modulus exhibited by P­(BS_20_BDG_80_) may raise considerations regarding mechanical tolerability during ocular application. Although this copolymer displays increased stiffness compared to P­(BS_50_BDG_50_), both materials maintain mechanical characteristics typical of soft polymeric systems intended for contact with delicate tissues.^61^ Notably, the high elongation at break values observed for both copolymers indicate substantial ductility, which may reduce localized mechanical stress and improve conformability to the ocular surface. In addition to mechanical behavior, surface wettability represents a key factor influencing ocular comfort and device retention.^62^ The moderate hydrophilicity exhibited by both copolymers after short-term exposure to aqueous environments may promote beneficial interactions with the tear film and mucin layer, potentially reducing friction during blinking and improving tolerability.^13,16,61,62^

### 3.3 Scanning Electron Microscopy (SEM)

SEM analysis was carried out on profiles and cross sections of the cylindrical devices obtained by both copolymers to confirm that the extrusion processing was successful. According to Figure 2, both cylinders were demonstrated to be defect-free, all the surfaces and sections were flat, smooth, and without any holes or cavities. These findings support the absence of phase separation lines in both materials.

**Figure 2:**
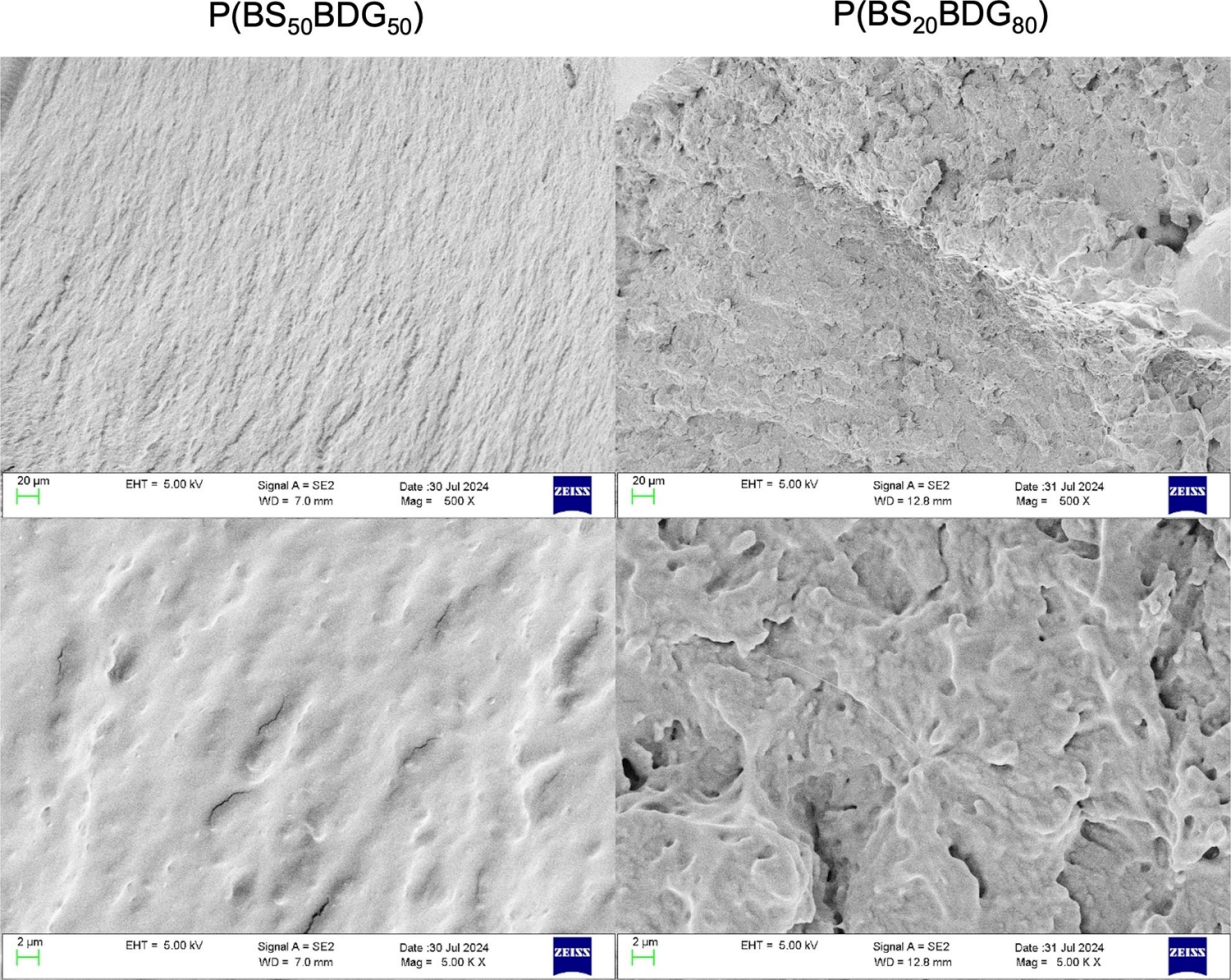
SEM pictures at 500× (top) and 5000× (bottom) magnifications of cryo-fractured sections of copolymeric cylinders.

The combined contribution of mechanical flexibility, surface wettability, and smooth defect-free morphology of the extruded cylinders is therefore expected to minimize foreign body sensation. However, the mechanical comfort of ocular inserts is influenced by multiple factors, including device geometry, tear film dynamics, and blinking-related mechanical forces. Therefore, dedicated *in vivo* tolerability and comfort studies will be required to fully evaluate the patient acceptability of the proposed delivery system.

### 3.4 Model Proteins Elution Test

#### 3.4.1 Lysozyme and Lactoferrin Elution on Melted Copolymers

Quantitative analysis of each protein was performed by automated electrophoresis, observing the cumulative release. No evidence of polymer swelling, structural alteration, or disintegration was observed after the 3 h immersion in PBS under the described conditions. The elution of proteins encapsulated by melting was observed, and results proved that cumulative release occurred at different time-points (Figure 3). It is evident from the data that elution of lysozyme and lactoferrin increased over time for both copolymers (Figure 3a,b). Lysozyme release occurred over a period of 120 min (Figure 3a), whereas for lactoferrin it was observed over a period of 320 min (Figure 3b). In the case of Lf, there is a delay time of 5 min for P­(BS_20_BDG_80_) and of 10 min for P­(BS_50_BDG_50_). Moreover, Lf was released with a lower cumulative concentration over time compared to Lys, and more rapidly for P­(BS_50_BDG_50_) compared to P­(BS_20_BDG_80_). All the observed differences may be attributed to the specific interactions between the model protein and each copolymer, taking into account their different hydrophilicity and also considering the steric effect due to higher molecular weight of Lf (∼80 kDa) compared to Lys (14.5 kDa). These long elution times suggest that this encapsulation technique is effective in blocking the molecules inside the polymeric matrix, thus ensuring a prolonged release over time.

**Figure 3:**
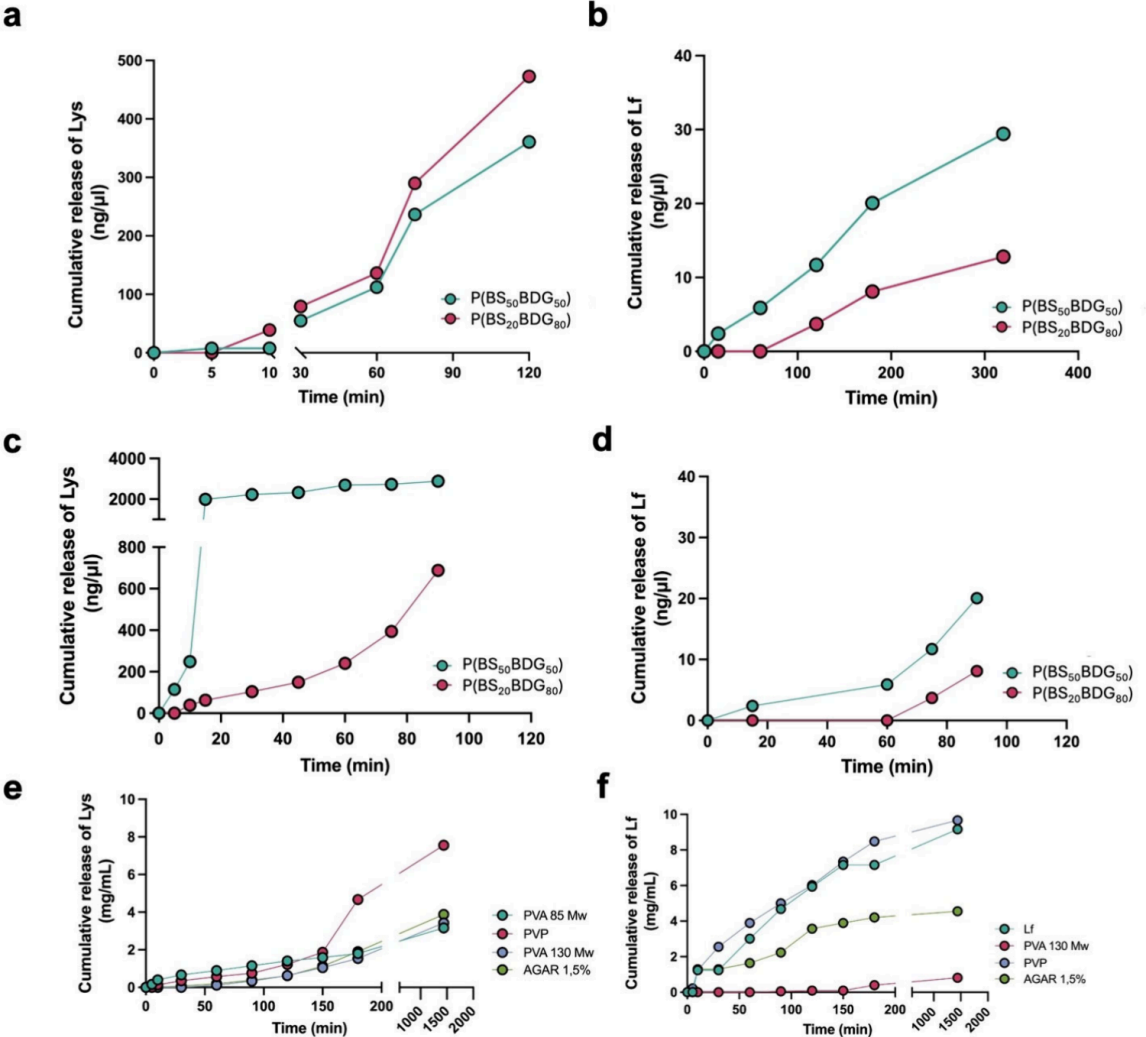
Time-dependent *in vitro* cumulative release postmelting of Lys (a) and Lf (b), postimmersion of disks in a solution of Lys (c) and Lf (d) expressed in ng/μL; and release of Lys (e) and Lf (f) from P­(BS_50_BDG_50_) differently coated cylinders expressed in mg/mL.

#### 3.4.2 Protein Elution after Immersion

In this analysis, Lys elution was constant already after 20 min for P­(BS_50_BDG_50_), while it increased over time for P­(BS_20_BDG_80_); P­(BS_50_BDG_50_) also eluted at a higher concentration of Lys (Figure 3c). As to Lf, also in this case, it was released later from both copolymers and with a lower cumulative concentration over time compared to Lys, probably due, once again, to its higher molecular weight. In particular, copolymer P­(BS_20_BDG_80_) started releasing Lf after 60 min (Figure 3d), probably due to its lower hydrophilicity compared to that of P­(BS_50_BDG_50_). Full protein release occurred in 100 min, for both copolymers, suggesting that this technique, compared to encapsulation through melting, is less effective in delaying the release of the proteins.

For ocular surface applications, release profiles were intentionally expressed as absolute protein concentrations, as therapeutic efficacy in dry eye disease is defined by drug concentration at the ocular surface rather than by the fraction of release from the delivery system. This approach allows a direct comparison with clinically relevant concentration ranges reported for topical ophthalmic therapies.

#### 3.4.3 Protein Elution from the Coating

P­(BS_50_BDG_50_) was selected to perform extrusion tests to form a cylindrical material as it had provided better results in terms of wettability and protein release characteristics. The polymers used for each outer layer, Poly-Vinyl-Pyrrolidone PVP, Poly-Vinyl-Alcohol PVA (*M*_w_: 85000 and 130000) and Agar (1,5%), showed an improvement on proteins leaching, especially by prolonging the time and the concentration released. The elution curves are shown in Figure 3 for the devices coated including lysozyme (Figure 3e) and lactoferrin (Figure 3f). Release analyses show that both copolymers sustain a gradual release of proteins over time within 24h. Furthermore, among the biomaterials used to cover the cylinders, PVA demonstrated the better retention of proteins over time. Further analyses incorporating different concentrations of each coating are ongoing in order to modify the loading and anchoring characteristics of the proteins. This will facilitate the modulation of concentration of the protein incapsulated and their release kinetics. In general, without the use of coating, the difference in the release kinetics is due to a combination of several factors, such as the distinct molecular weight and steric size of each protein,^26,63,64^ both higher for lactoferrin, as well as the higher hydrophobicity of P­(BS_20_BDG_80_) compared to P­(BS_50_BDG_50_). However, both copolymers can be considered potentially suitable for releasing active molecules even over long periods of time. Although the *in vitro* release experiments were performed under static conditions, the obtained release profiles can be interpreted considering the highly dynamic nature of the human tear film. Tear turnover (0.5–2.2 μL/min) is a major determinant of ocular drug clearance and contributes to the low bioavailability of conventional ophthalmic formulations.^65,66^ In this context, the burst release observed after thermally induced melting at physiological temperature (37 °C) may represent a therapeutically advantageous feature, enabling rapid delivery of a pharmacologically relevant protein dose within the short precorneal residence time. Similar early release profiles have been reported as beneficial in ocular drug delivery systems designed to compensate for tear turnover and blinking-related clearance mechanisms.^62,65,66^ Moreover, the thermoresponsive behavior of P­(BSxBDGy) copolymers may enhance conformational adaptation and contact with the ocular surface, potentially improving local retention compared to conventional eye drops, particularly when moderate wettability and mechanical flexibility promote effective interaction with mucin layers and conjunctival tissues.^13,15^ Nevertheless, static immersion models cannot fully replicate the complexity of the ocular surface environment; therefore, dynamic in vitro models and in vivo studies will be required to further assess translational performance under physiologically relevant tear turnover conditions.

### 3.5 Circular Dichroism (CD)

The structural conformation of C-lysozyme (C-Lys) and lactoferrin (Lf) was evaluated by CD spectroscopy before and after their release from both (Figure 4). CD spectra of C-Lys displayed characteristic features of α-helix and β-sheet structures, with decreased ellipticity at 208 and 230 nm in the far-UV region (Figure 4a). In contrast, the CD spectra of Lf exhibited two minima at 208 and 220 nm, indicative of a predominantly α-helical conformation (Figure 4b). Comparison of spectra obtained before and after release revealed no appreciable differences for either protein, demonstrating that neither copolymer A nor copolymer B altered the native secondary structure of C-lysozyme or lactoferrin.^59^

**Figure 4:**
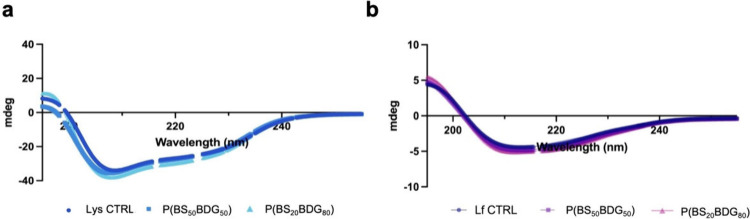
CD spectra of (a) human C-Lysozyme (C-Lys) and (b) lactoferrin (Lf) observed following the release from copolymers.

### 3.6 Biocompatibility Evaluation

#### 3.6.1 Direct Contact Test

Data obtained from the cell viability assay on HConF (Figure 5a) and on HConEpiC (Figure 5b) indicate that cell viability was not modified after 24 h as compared to the control. Subsequently, graphs demonstrate a progressive increase of vitality at 48 and 72 h. Therefore, the present study confirms that both copolymers do not show any cytotoxic effects.

**Figure 5:**
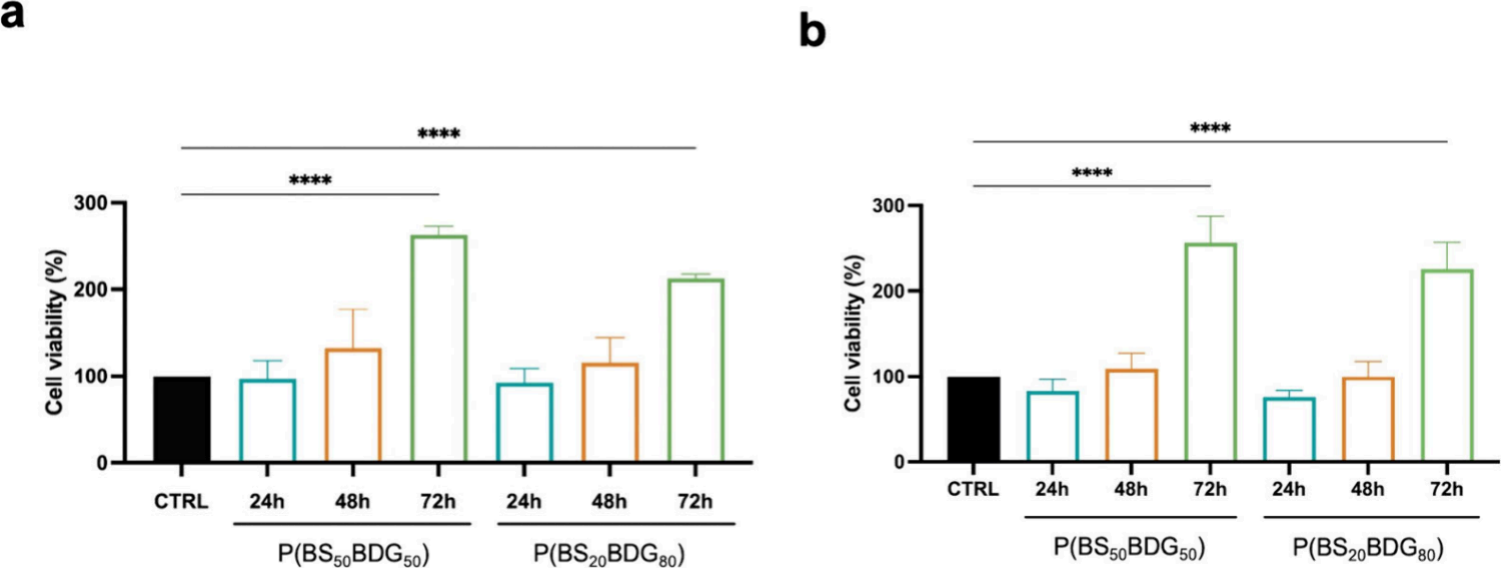
Cytotoxicity test suggested by ISO 10993-5. Direct contact test of both polymers with (a) human conjunctival fibroblast and (b) conjunctival epithelial cells (ATCC) performed with the MTT test.

#### 3.6.2 Test on Extracts

Cells were exposed to copolymer extracts to evaluate the release of the toxic substances. The cytocompatibility of both copolymers was tested through the MTT assay with HConF (Figure 6a–c) and HConEpiC (Figure 6c–e). The test was done using 100%, 50%, and 25% extracts that were taken after 24, 48, and 72 h of incubation. As can be seen in Figure 6, all the experimental groups showed more than 70% cell viability in comparison to the control, thereby showing no cytotoxic effects. The HConF and HConEpiC viability exhibited by P­(BS_50_BDG_50_) was demonstrated to be time-dependent, with an increase observed at the 48 h time point. This was further substantiated by the optical density (O.D.) values obtained from the 50% (Figure 6b) and 25% of extracts for HConF (Figure 6c) and for all the HConEpiC extract ratios, which exceeded the control (Figure 6c–e). A small but significant increase in cell density was observed in P­(BS_20_BDG_80_) extracts O.D. values at 48 h (Figure 6c), at 72 h (Figure 6b) and in P­(BS_50_BDG_50_) extracts at 48 h (Figure 6b,c). These findings suggest that the 100% extract is nontoxic for HConF but instead showed an increase in cellular metabolic activity.

**Figure 6:**
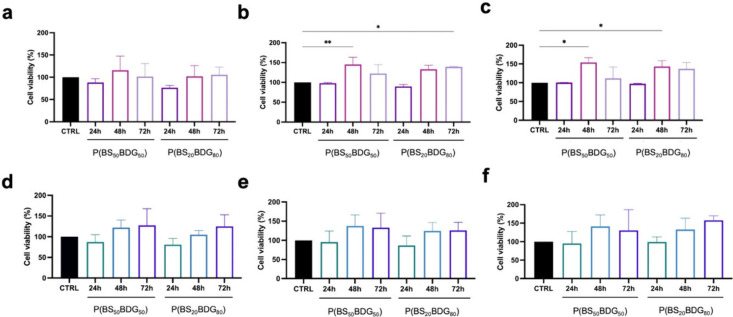
Cytotoxicity test suggested by ISO 10993-5. Test on extract on HConF using copolymers extract ratio at (a) 100%, (b) 50%, and (c) 25% and on HConEpiC cultured with copolymers elution ratio of (d) 100%, (e) 50%, and (f) 25%. Statistical significance was assessed using a one-way analysis of variance (ANOVA) with Tukey’s post hoc test. *P < 0.05, **P < 0.01.

Differences between extract-based and direct contact assays at later time points likely reflect variations in cellular metabolic adaptation and physical cell–material interactions rather than cytotoxic effects, as MTT readouts primarily indicate mitochondrial activity.

#### 3.6.3 Morphology Evaluation

The microscopic evaluation of both HConF (Figure 7) and HConEpiC (Figure 8) cultured in direct contact with both copolymers (red arrows) reveals no appreciable differences between cells exposed only to a basic medium (CTRL).

**Figure 7:**
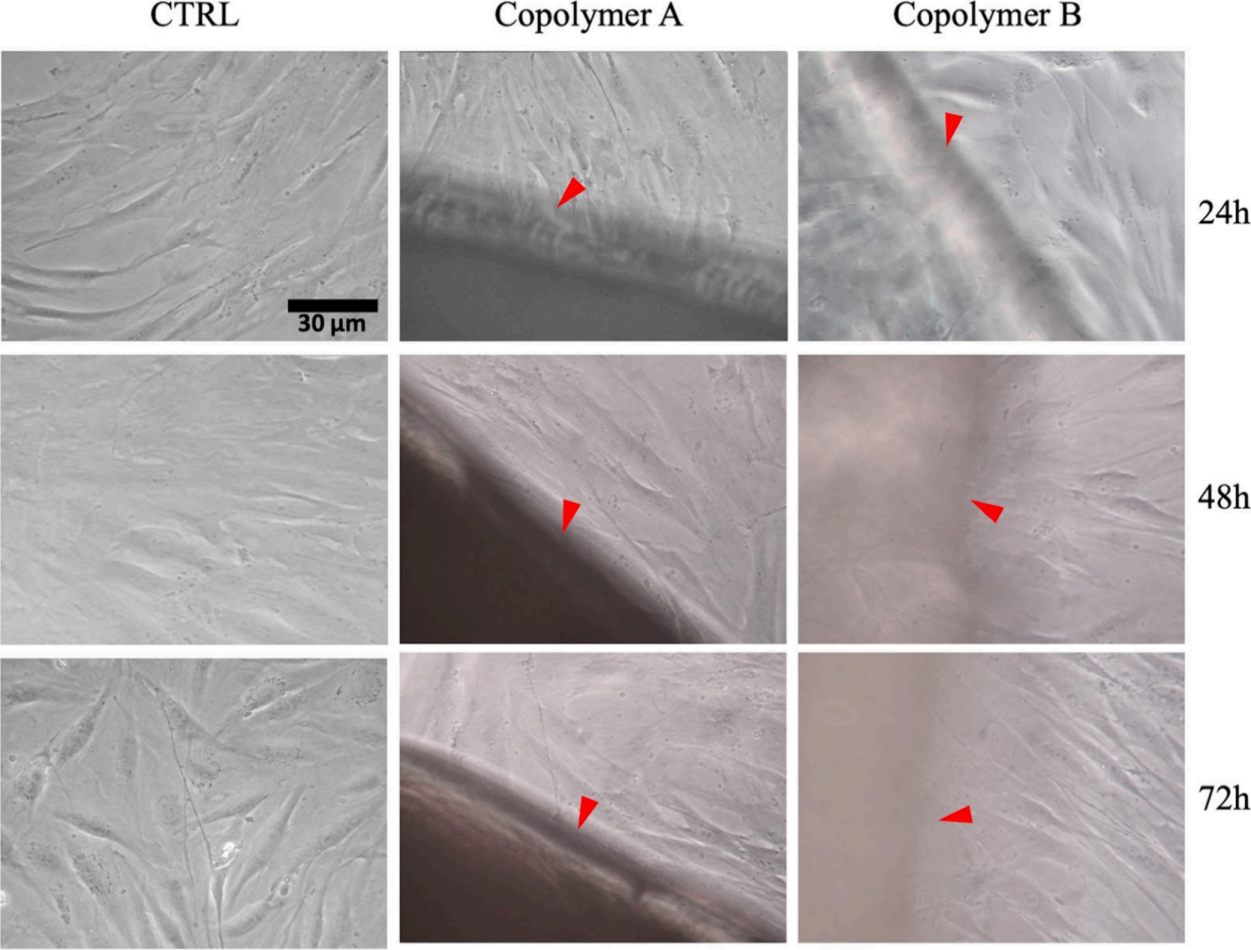
Morphology evaluation of HConF grown in direct contact with P­(BS_20_BDG_80_) and P­(BS_50_BDG_50_) by optical microscopy. After 24, 48, and 72 h of culture no significant morphological differences were found between polymer-free cells (CTRL) and cells cultured in the presence of copolymers, original magnification 20×.

**Figure 8:**
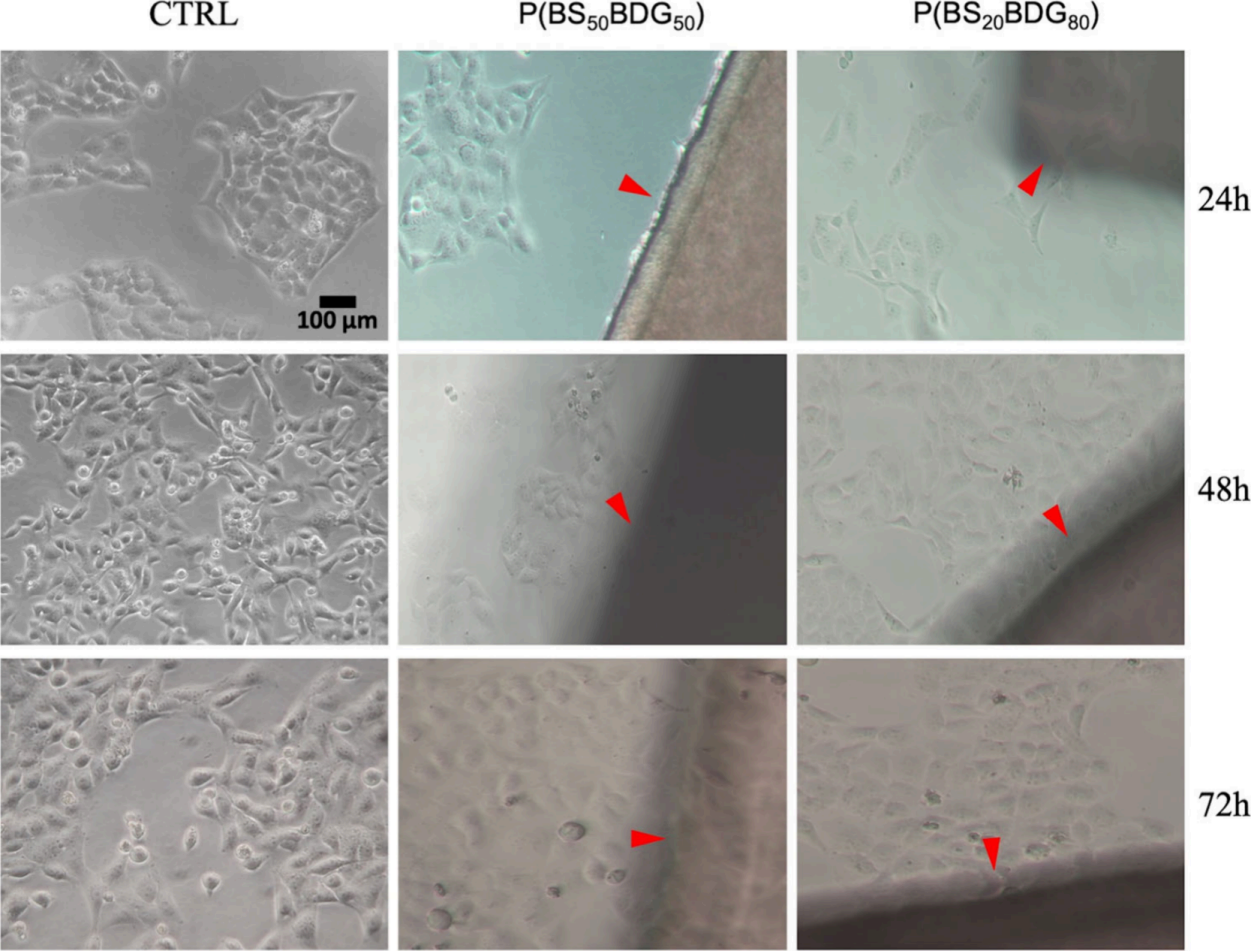
Morphology evaluation of HConEpiC grown in direct contact with P­(BS_20_BDG_80_) and P­(BS_50_BDG_50_) by optical microscopy. After 24, 48, and 72 h of culture no significant morphological differences were found between polymer-free cells (CTRL) and cells cultured in the presence of copolymers, original magnification 10×.

## 4 Conclusion

In the present study, two aliphatic random copolymers were synthesized by introducing different amounts of ether oxygen-containing BDG counits along the PBS macromolecular chain, tailoring their mechanical flexibility and wettability. The polymers were synthesized via a solvent-free, scalable method, yielding high-molecular-weight materials, and were successfully extruded into small cylindrical devices. As a result, their thermal, mechanical, and surface wettability properties were finely tuned as a function of their chemical composition. The potential of both copolymers for ocular drug delivery was confirmed, as a sustained, composition-dependent release of lysozyme and lactoferrin, which were chosen as model tear proteins, was observed. Moreover, circular dichroism spectroscopy confirmed that both lysozyme and lactoferrin retained their native secondary structures during incubation and release from copolymers. Importantly, the polymeric coatings embedding the model proteins simultaneously enabled a prolonged and controlled release profile over time. This sustained release could be effectively modulated by varying the molecular weight of the polymers composing the coating layer, highlighting the tunability of the drug-delivery system. Extract-based and direct contact assays on human conjunctival cells showed no cytotoxicity and cell morphology similar to that of untreated controls. Thus, all of these findings highlight the suitability of both copolymers for controlled ophthalmic drug delivery.

## Supplementary Material



## Notes

Additional physicochemical characterization of the copolymers including specifically ^1^H NMR spectra (Figure S1), TGA curves (Figure S2), DSC scans (Figure S3), and stress–strain curves (Figure S4) of P­(BS_50_BDG_50_) and P­(BS_20_BDG_80_) copolymers (PDF)
