# Supplementary material for: Random Copolyester-Based Delivery Systems for Tear Protein Therapeutics in Ocular Surface Disorders
Source: ACS Omega. 2026 May 18;11(21):30390–402. doi: 10.1021/acsomega.5c09194 (PMC13234661; doi:10.1021/acsomega.5c09194)
Supplement: Supplementary file 1 [file ao5c09194_si_001.pdf]

Random Copolyester-Based Delivery Systems for Tear Protein Therapeutics in Ocular Surface Disorders

Gloria Astolfi[1], Giulia Guidotti[2], Michelina Soccio[2], Franco Dominici[3], Debora Puglia[3], Nadia Lotti[2], Erika Ponzini[4], Silvia Tavazzi[4], Piera Versura[1,5*], Luigi Fontana[1,5]

1. Ophthalmology Unit, DIMEC, Alma Mater Studiorum Università di Bologna, 40138, Italy; gloria.astolfi2@unibo.it; piera.versura@unibo.it; luigi.fontana6@unibo.it:
2. Department of Civil, Chemical, Environmental and Materials Engineering, Alma Mater Studiorum Università di Bologna, 40131, Italy; giulia.guidotti9@unibo.it; m.soccio@unibo.it; nadia.lotti@unibo.it
3. Department of Civil and Environmental Engineering, University of Perugia, 05100, Terni, Italy; franco.dominici@unipg.it; debora.puglia@unipg.it
4. Department of Materials Science, University of Milano-Bicocca, 20126, Milan, Italy; erika.ponzini@unimib.it; silvia.tavazzi@unimib.it
5. IRCCS Azienda Ospedaliero-Universitaria di Bologna, 40138, Italy

Corresponding author

Prof Piera Versura

Ophthalmology Unit, DIMEC - Alma Mater Studiorum University of Bologna

IRCCS Azienda Ospedaliero-Universitaria di Bologna

Via Palagi, 9 I-40138 Bologna phone +39-051-2142850

piera.versura@unibo.it

SUPPORTING INFORMATION

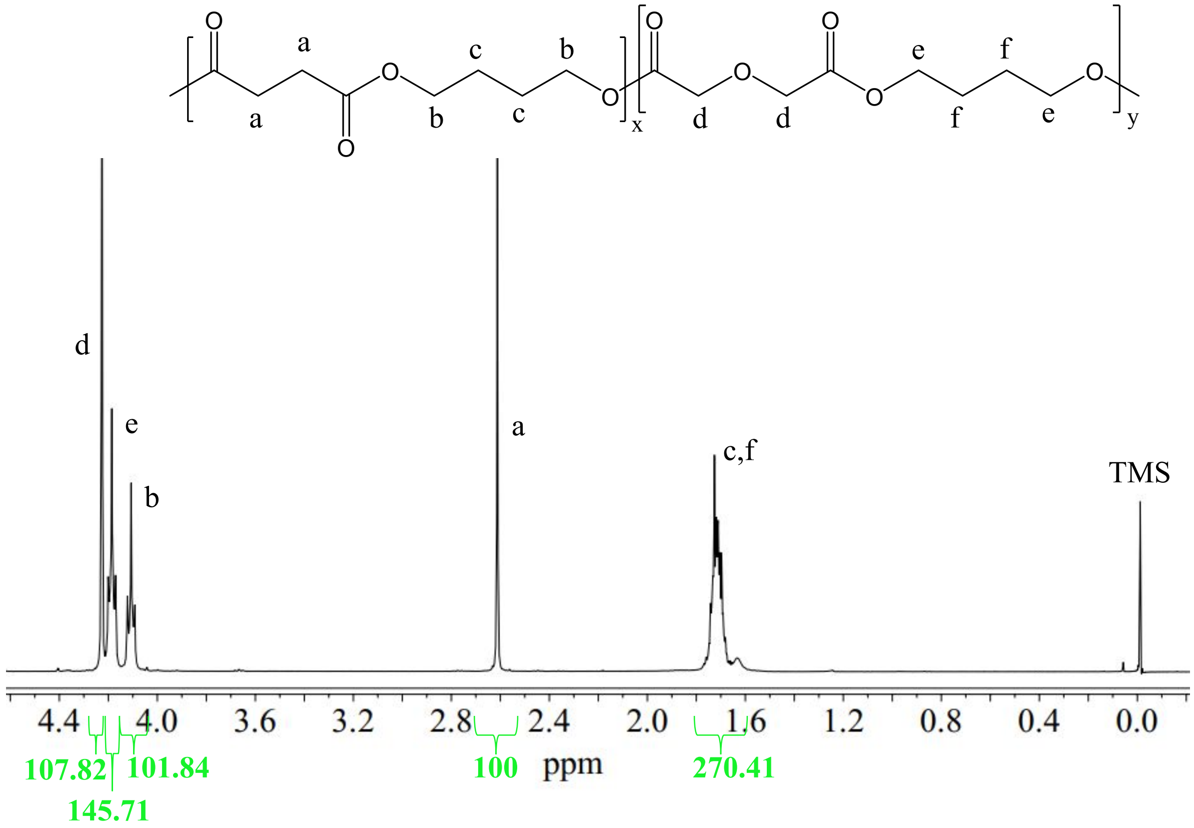

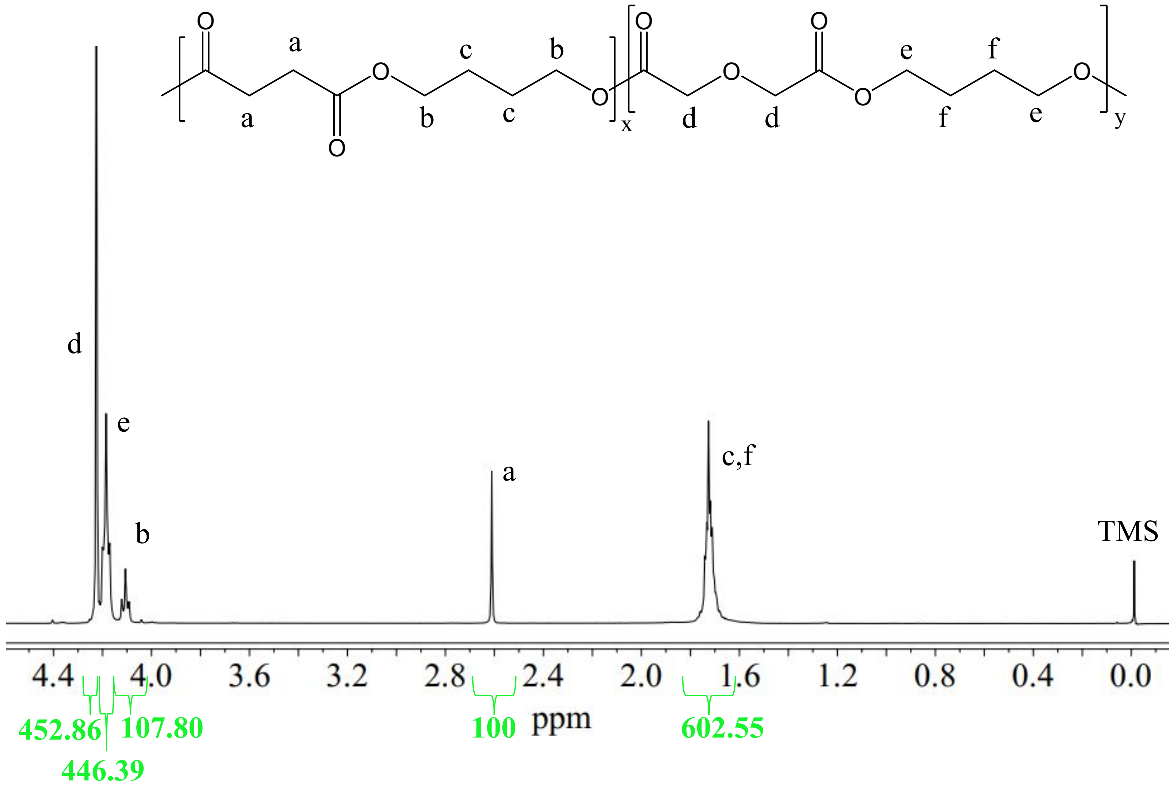

Figure S1: ^{1}H-NMR spectra of P($BS_{50}BDG_{50}$) (top) and P($BS_{20}BDG_{80}$) (bottom) copolymers.

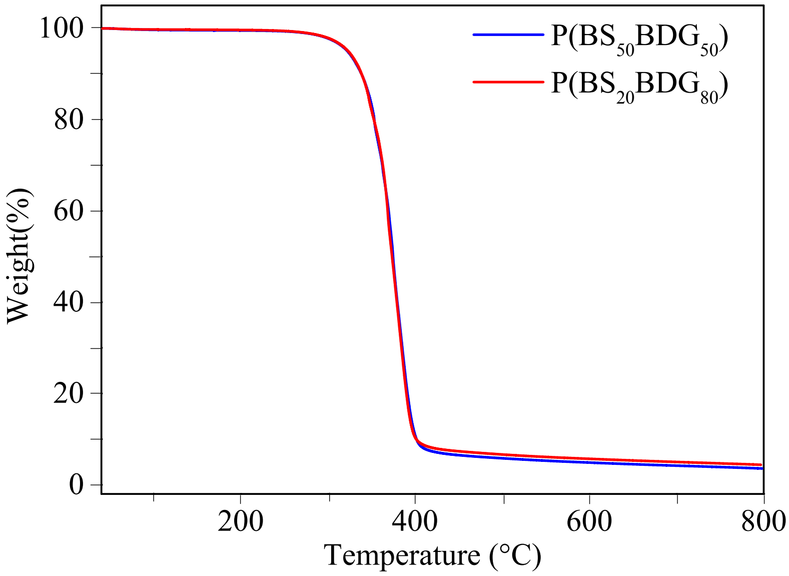

Figure S2: TGA curves of P($BS_{50}BDG_{50}$) and P($BS_{20}BDG_{80}$) copolymers.

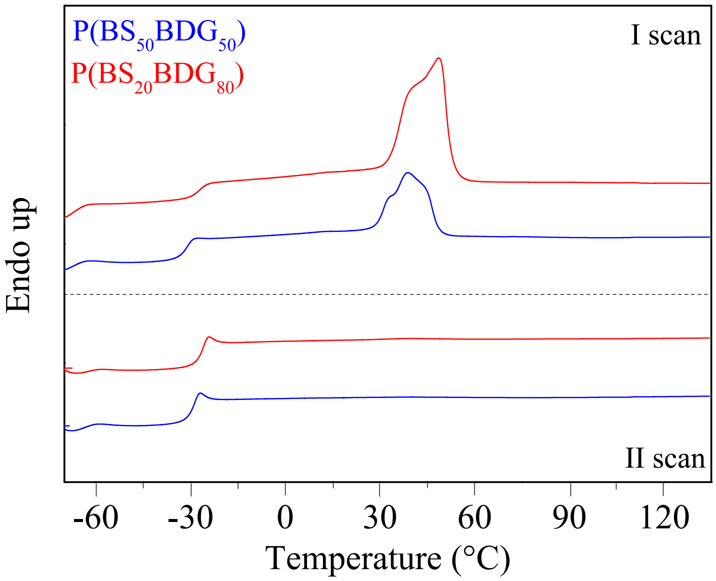

Figure S3: I and II scan DSC curves of P($BS_{50}BDG_{50}$) and P($BS_{20}BDG_{80}$) copolymeric films.

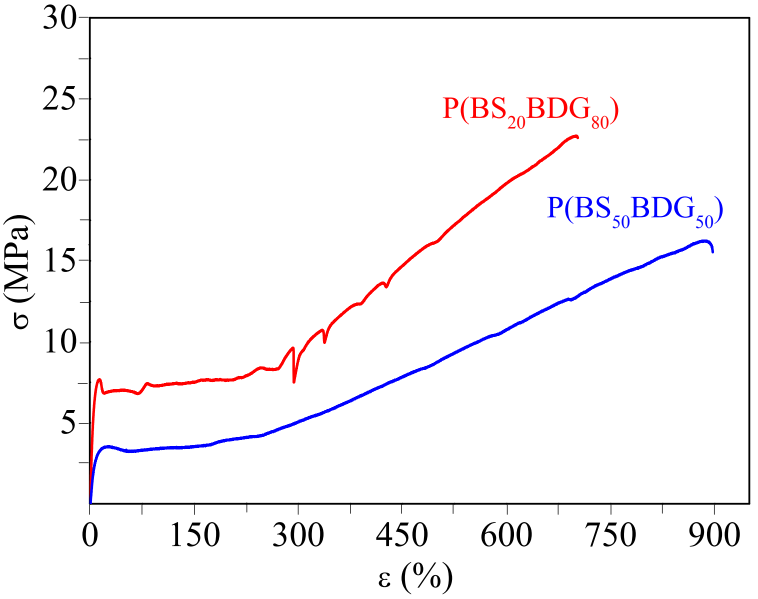

Figure S4: Stress-strain curves obtained from films of $P(BS_{50}BDG_{50})$ and $P(BS_{20}BDG_{80})$ copolymers.
